# Arthroscopy or arthrotomy for native knee septic arthritis: A systematic review

**DOI:** 10.1002/jeo2.12041

**Published:** 2024-06-06

**Authors:** Daniel P. McKenna, Peggy Miller, Timothy McAleese, May Cleary

**Affiliations:** ^1^ Department of Trauma and Orthopaedic Surgery University Hospital Waterford Waterford Ireland; ^2^ Department of Orthopaedic Surgery University College Cork Cork Ireland

**Keywords:** arthritis, knee, native, septic, washout

## Abstract

**Purpose:**

Septic arthritis of any joint is an orthopaedic emergency which requires prompt diagnosis and treatment. The knee is the commonest joint afflicted, and the primary objective of any treatment is complete source control. This commonly takes the form of antibiotic therapy and a washout of the infected joint by means of arthroscopy or arthrotomy. The primary aim of this review is to investigate if arthroscopic washout for native knee septic arthritis confers a lower risk of repeat procedure than arthrotomy.

**Methods:**

A systematic review and meta‐analysis was conducted of the MEDLINE, SCOPUS and the Cochrane Library data bases. The primary outcome of interest was requirement for repeat washout with all‐cause complications, length of inpatient stay and mortality secondary outcomes.

**Results:**

A total of 17,140 subjects were included for analysis of the primary outcome, and the overall rate of repeat procedure was 14.6%. No statistical difference was found between arthroscopy and arthrotomy for repeat washout (risk ratio 0.86 [95% confidence interval, CI: 0.72–1.02], *I* ^2^ = 36%). Eligible studies found in favour of arthroscopy for all‐cause complication rate (risk ratio 0.75 [95% CI: 0.6–0.93], *I* ^2^ = 84%) and length of stay in hospital (mean difference −1.98 days [95% CI: −3.43 to −0.53], *I* ^2^ = 84%). No statistical difference was found for the mortality rate (risk ratio 1.17 [95% CI: 0.52–2.63], *I* ^2^ = 57%).

**Conclusion:**

Our analysis found arthroscopy and open arthrotomy to be equivocal for repeat surgical washout in native knee septic arthritis. All‐cause complication rate and length of inpatient stay were favourable for arthroscopy with no difference noted between mortality rates.

**Level of Evidence:**

Level III.

**Abbreviations**
ICD: International Classification of Diseases
MeSH: medical subject headings
NRD: National Readmission Database
NSQIP: National Surgical Quality Improvement Programme
PRISMA: Preferred Reporting Items for Systematic Reviews and Meta‐analyses
RCT: randomised controlled trials
ROBINS‐I: Risk of Bias in Nonrandomised Studies‐of Interventions
WBC: white blood cell

## BACKGROUND

Septic arthritis of any joint constitutes an orthopaedic emergency, necessitating immediate diagnosis and intervention [17]. The knee is the most frequently affected joint, predominantly by *Staphylococcus aureus* infection [31, 34]. Septic arthritis is associated with significant morbidity, evolving quickly to osteomyelitis, joint cartilage destruction and septicaemia. It is also associated with a 30‐day mortality risk between 1% and 9% [3, 9, 21]. Those most at risk of mortality are elderly patients and those with diabetes and renal failure [31].

The gold standard diagnostic method for septic arthritis in the acute setting is joint aspiration [29]. Samples with a white blood cell (WBC) count exceeding 50 × 10^9^/L are considered diagnostic, however, other investigations such as gram staining and culture are essential for a comprehensive clinical understanding [8]. Of note, the administration of antibiotic therapy prior to aspiration is associated with a decrease in synovial leucocyte count [32].

The fundamental objective of treatment is to achieve complete source control. This typically involves the use of antibiotic therapy and a surgical washout of the infected joint. Prompt administration of intravenous antibiotics is recommended postaspiration of the joint in question [33]. Delayed antibiotic cover is associated with extended antibiotic therapy and postponed de‐escalation [4]. Empiric antibiotic therapy is directed by local guidelines with usual options including cephalosporins, vancomycin and aminoglycosides [16, 20].

The surgical washout of a joint can either take the form of arthroscopy or arthrotomy. Contradictory conclusions have been drawn from prior reviews regarding the efficacy of arthroscopy in eradicating infection [2, 28]. There is also no consensus on the functional and clinical results associated with both procedures for various patient cohorts.

Given the persistent uncertainty, the emergence of new evidence on this topic and the prevalence of knee involvement in septic arthritis, this research was conducted to identify the most effective method of surgical washout. This review focuses on native knee joint septic arthritis only in order to minimise confounding factors.

The primary aim of this review is to discern if arthroscopic washout for native knee septic arthritis confers a lower risk of repeat procedure compared to arthrotomy. This study defines a successful knee washout as an infection treated with one procedure and deems the need for multiple washouts as treatment failure. Our secondary outcome was to compare the clinical outcomes of both procedures with respect to complication rates, hospital length of stay and mortality.

## METHODS

### Registration

This trial was registered with PROSPERO, and the search strategy was carried out in accordance with the Preferred Reporting Items for Systematic Reviews and Meta‐analyses (PRISMA) guidelines [40].

### Eligibility criteria

Inclusion criteria for this review were limited to randomised controlled trials (RCT), case control and cohort studies comparing arthroscopy and arthrotomy for the management of native knee septic arthritis. Studies had to include subjects aged strictly 18 years of age and over. Only original studies reported in English and those published in peer‐reviewed journals in the last 10 years were eligible for inclusion. No restriction was placed on the treatment setting or the geographical location of the subjects. No limitation was placed on the length of follow‐up following the original washout.

Case series and case reports were excluded. All studies involving paediatric patients and joints with a prosthesis were excluded. Any descriptive studies not providing statistical analysis were excluded.

### Diagnosis and outcomes

A diagnosis of native knee septic arthritis was based on clinical examination, joint aspiration, correlation with a clinical grading system or as classified by the International Classification of Diseases. Studies directly comparing outcomes post washout by means of arthroscopy or arthrotomy were selected. The primary outcome of interest was requirement for repeat operative intervention. Secondary outcomes included all‐cause complication rate, length of stay and mortality.

### Search strategy

A search strategy using medical subject headings (MeSH) relating to septic arthritis was devised. MEDLINE, SCOPUS and the Cochrane Library were searched between 1 January 2013 and 31 July 2023. For completeness, on‐going trials were searched through the International Clinical Trials Registry Platform search portal and systematic reviews on the topic were searched using the PROSPERO register. The data bases and their corresponding search inputs are outlined in Table 1.

**Table 1:** Search strategy.

Data base	Search terms		
MEDLINE	((((“Arthroscopy”) OR (arthroscopy or arthroscopy)) AND (open or arthrotomy)) AND ((knee) OR (Knee))) AND ((Arthritis, Infectious) OR (septic arthritis OR septic OR sepsis OR Infective OR infection))		
SCOPUS	TITLE‐ABS‐KEY (((arthroscopy OR arthroscopy) AND (open OR arthrotomy)) AND (knee) AND ((arthritis OR septic AND arthritis OR septic OR sepsis OR infective OR infection))		
Cochrane library	#1	MeSH descriptor: explode all trees	1980
	#2	MeSH descriptor: [Knee] explode all trees	1593
	#3	MeSH descriptor: [Arthritis, Infectious] explode all trees	135
	#4	((arthroscopy or arthroscopy) AND (open or arthrotomy)):ti,ab,kw	370
	#5	(knee):ti,ab,kw	38999
	#6	((septic arthritis OR septic OR sepsis OR Infective OR infection)):ti,ab,kw	159483
	#7	#1 AND #4	
	#8	#2 AND #5	
	#9	#3 AND #6	
	#10	#7 AND #8 AND #9	

### Data management

Two independent reviewers (D. P. M. K. and P. M.) employed the above strategy. Following identification of suitable studies, predetermined descriptive data were recorded for each included article. The findings from each reviewer were compared and any conflicts were independently resolved by a third reviewer (T. M. A.). Accuracy of data extraction was ensured through cross‐checking of the predetermined data points. Authorship and sample numbers were used to identify the possibility of overlapping or companion studies.

A risk of bias assessment was carried out for each included study. This was completed using the Risk of Bias in Nonrandomised Studies of Interventions (ROBINS‐I) tool [43]. This tool was employed by the two independent reviewers. Disagreements were resolved where possible, and input from the third independent reviewer was sought as necessary.

### Data analysis

Data management and analysis were conducted through RevMan 5.6 [44]. Dichotomous data were analysed by using risk ratios with the confidence interval (CI) set at 95%. For continuous data, a mean difference was calculated, again a 95% CI was used. Heterogenicity was assessed using the *χ* ^2^ test and *I* ^2^ statistic with >50% deemed as significant heterogenicity. For data without significant heterogenicity, the Mantel–Haenszel fixed effect model was used. A random effects model was chosen for data with significant heterogenicity. In order to examine the influence of each individual study, the leave‐one‐out method was used during analysis of the primary outcome.

## RESULTS

### Articles selected for inclusion

**Figure 1:**
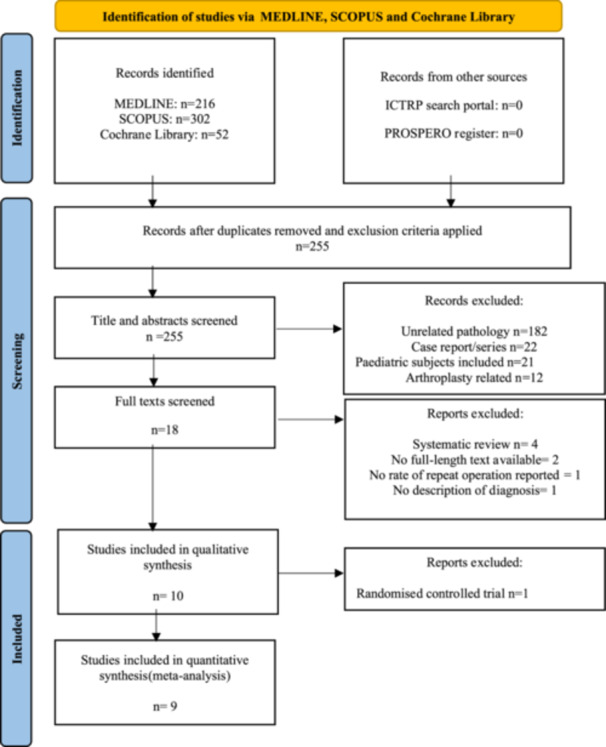
Preferred Reporting Items for Systematic Reviews and Meta‐analyses flow diagram outlining the search strategy and corresponding results.

A total of 570 studies were identified following an initial search of the three selected data bases. The exclusion criteria were applied and duplicates were removed leaving 255 articles. The title and abstract of each were reviewed and those deemed ineligible were accordingly excluded. The primary indications for exclusion were studies examining unrelated pathology, such as infection post anterior cruciate ligament repair, and case reports. Full texts of 18 articles were reviewed with nine deemed eligible for inclusion in the final analysis [6, 7, 10, 14, 23, 24, 25, 42, 45]. A full description of the selection process is outlined in the PRISMA flow diagram [36] (Figure 1). Baseline characteristics of each article were recorded in the standardised data collection document (Table 2). Available subject demographics are displayed in Table 3.

**Table 2:** Study baseline characteristics.

Authors	Year	Subject recruitment and timeframe	Diagnosis	Number of subjects	Length of follow‐up, months
Bohler	2015	Single institution 2002–2010	Aspiration, blood culture	70	3
Bovonratwet	2017	NSQIP 2005–2014	ICD 9	384	1
Dave	2015	Single institution 1995–2011	History, clinical exam, aspiration	52	194
Faour	2019	NSQIP 2011–2015	ICD 9	695	1
Jaffe	2016	Single institution 2000–2016	Hospital billing data base	80	4
Johns	2017	Single institution 2000–2015	Newman grade	161	5.3
Johnson	2020	NSQIP 2006–2016	ICD 9	1270	1
Stake	2019	Single institution 2005–2015	Aspiration	63	Not reported
Upfill‐Brown	2022	NRD 2016–2019	ICD 10	14,365	6

Abbreviations: ICD, International Classification of Diseases; NRD, National Readmission Data base; NSQIP, National Surgical Quality Improvement Programme.

**Table 3:** Subject demographics.

Authors		Age (years)	Male gender (%)	BMI	Diabetes (%)	Smoking (%)
Bohler		Median		Median		Not reported
	Arthroscopy	49	65.9	25.4	17.1	
	Arthrotomy	71	65.5	27.2	34.5	
Bovonratwet		Mean			Not reported	Not reported
	Arthroscopy	60	67.6	29		
	Arthrotomy	58	66.7	31		
Dave		Not reported	Not reported	Not reported	Not reported	Not reported
	Arthroscopy					
	Arthrotomy					
Faour		Mean		Median	Not reported	
	Arthroscopy	60	65	29.2		23
	Arthrotomy	58	65	31.2		26
Jaffe		Method not stated		Not stated		
	Arthroscopy	59.6	66.7	28.8	33.3	30.3
	Arthrotomy	47.3	55.3	27.7	23.4	56.3
Johns		Median		Not reported		
	Arthroscopy	57.5	65		13	31
	Arthrotomy	65.8	65		19	40
Johnson		Median		Not reported	Not reported	
	Arthroscopy	57.4	66			23.3
	Arthrotomy	57.3	68			29.1
Stake		Not reported	Not reported	Not reported	Not reported	Not reported
	Arthroscopy					
	Arthrotomy					
Upfill‐Brown		Median		Not reported	Not reported	Not reported
	Arthroscopy	57.3	66.2			
	Arthrotomy	57.1	66.4			

### Risk of bias analysis

**Figure 2:**
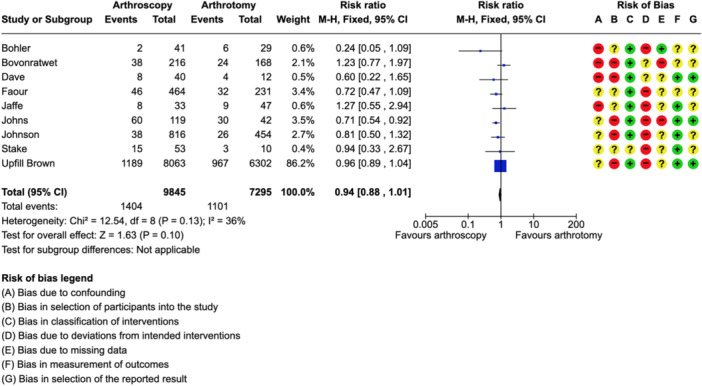
Forest plot depicting requirement for repeat washout in the nine included studies. CI, confidence interval; M‐H, Mantel‐Haenszel.

The ROBINS‐I tool was applied to each of the nine included studies. All studies were found to have a serious risk of bias in at least one domain. This bias predominantly stems from the retrospective and nonrandomised nature of the included articles, specifically in terms of deviations from intended interventions and subject selection. Intended interventions were largely surgeon‐dependent or the rationale for choice of washout modality was not clearly stated. Moreover, subjects were recruited from single‐centre or national data bases, placing reliance on accurate data entry and coding. Despite these issues, the objective measurement of the frequency of repeat washout, a target outcome for all of the included studies remained uncompromised. Overall, a moderate risk of bias was observed in the included articles, offering reliable evidence. A visual depiction of the risk of bias is shown in Figure 2.

### Requirement for repeat washout

A total of 17,140 subjects were included in the analysis focusing on the primary outcome of repeat washout. The overall rate of repeat procedure was 14.6%. A comparative assessment between arthroscopy and arthrotomy demonstrated no statistically significant difference (risk ratio 0.86 [95% CI: 0.72–1.02]), Figure 2. The *I* ^2^ = 36% indicates low heterogenicity, therefore, a fixed‐effects model was used.

In the leave‐one‐out sensitivity analysis, exclusion of Upfill‐Brown et al. [45] was the only article to give a statistically significant result. This exclusion changed the risk ratio to 0.82, in favour of arthroscopy ([95% CI: 0.68–0.99], *p* = 0.004). Table 4 gives a full description of the results with each individual study omitted.

**Table 4:** Leave‐one‐out analysis.

Omitted study	Risk ratio, (95% confidence interval)	*p* Value	*I* ^2^ (%)
Bohler	0.95 (0.88–1.02)	0.13	26
Bovonratwet	0.94 (0.87–1.01)	0.13	37
Dave	0.94 (0.88–1.01)	0.11	41
Faour	0.95 (0.88–1.02)	0.17	37
Jaffe	0.94 (0.87–1.01)	0.1	42
Johns	0.95 (0.88–1.02)	0.18	10
Johnson	0.95 (0.88–1.02)	0.09	43
Stake	0.94 (0.88–1.01)	0.1	44
Upfill‐Brown	0.82 (0.68–0.99)	0.04	18

### All‐cause complication rate

**Figure 3:**
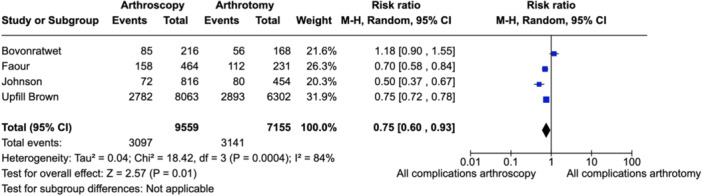
Forest plot depicting all‐cause complications rate in the four studies with data available. CI, confidence interval.

All‐cause complication rate was the key secondary outcome of interest. Four studies provided data for this analysis. Given *I* ^2^ = 84%, a random effects model was chosen. Arthroscopy was found to have a decreased incidence in all‐cause complication in comparison to arthrotomy (risk ratio 0.75 [95% CI: 0.6–0.93]) (Figure 3).

### Length of stay

**Figure 4:**
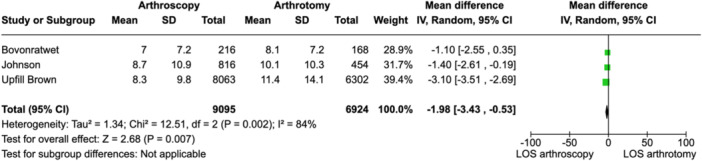
Forest plot depicting length of stay in the three studies with data available. CI, confidence interval.

The time spent as an inpatient was reported for five articles. However, no standard deviation was reported for two of these articles [6, 24], deeming them ineligible for inclusion in statistical analysis. Of the three studies with published standard deviations, arthroscopy had a statistically significant decrease in the average length of inpatient stay compared to arthrotomy (mean difference −1.98 days [95% CI: −3.43 to −0.53], *I* ^2^ = 84%) (Figure 4).

### Mortality

**Figure 5:**
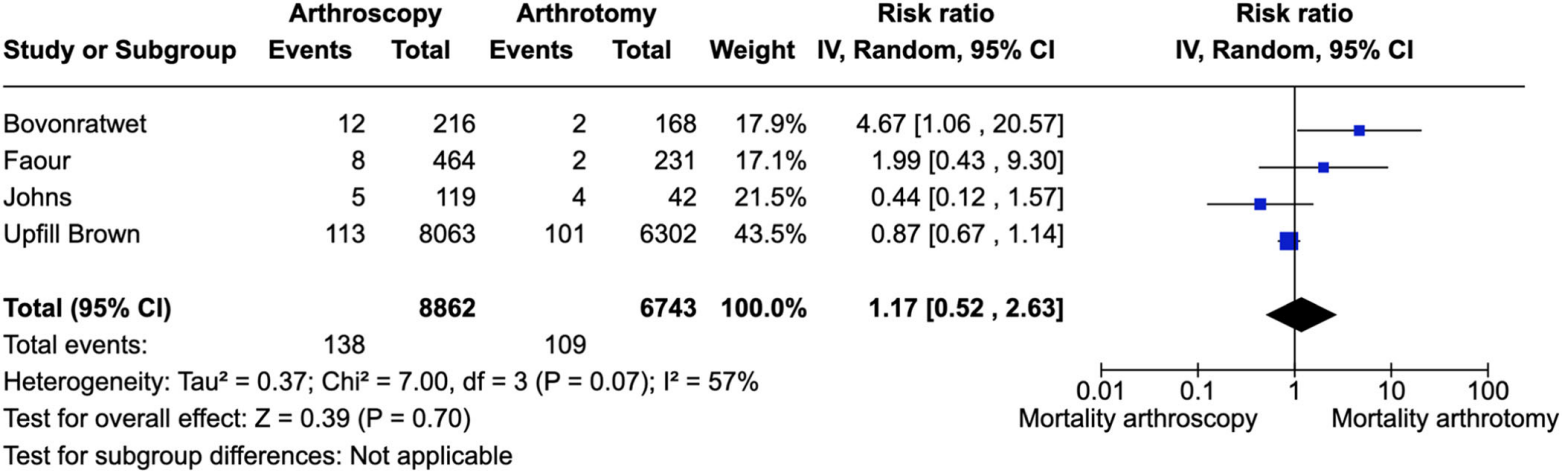
Forest plot depicting mortality in the four studies with data available. CI, confidence interval.

Four studies reported on mortality with the overall rate of 1.6%. There was no statistical difference between the mortality rate in the arthroscopy subjects compared to those undergoing arthrotomy (risk ratio 1.17 [95% CI: 0.52–2.63], *I* ^2^ = 57%) (Figure 5).

## DISCUSSION

### Requirement for repeat washout and source control

The primary aim of this review was to determine the optimal surgical approach for native knee septic arthritis. Our unadjusted analysis found no statistically significant difference between arthroscopy and arthrotomy amongst the 17,140 subjects included in this review. The subsequent sensitivity analysis found the exclusion of Upfill‐Brown et al. [45] to give a significant risk ratio in favour of arthroscopy. Therefore, the overall conclusion of this review is that neither arthroscopy nor arthrotomy showed superiority in reducing reoperation rate. This aligns with findings of previous reviews of this subject [11, 28, 38] with the exception of Acosta‐Olivo et al. [2] who found that arthroscopic washout was superior.

Surrounding the decision‐making process for arthroscopic versus open arthrotomy washout, there are two different hypotheses. Advocates for arthroscopic washout contend that by maintaining a closed space for irrigation, it allows for fluid penetration throughout the joint capsule along both the medial and lateral joint gutters. This allows for greater soft tissue integrity and in theory confers improved function postoperatively. Clinicians in favour of open surgery propose that the improved surgical exposure is required for thorough debridement of soft tissues and bone if this is deemed necessary. However, focused evidence in the literature to substantiate these hypotheses remains scarce.

The main aim of joint washout, be it arthroscopy or arthrotomy, is complete source control. The source can range from direct inoculation, iatrogenic seeding [41], haematogenous spread and direct spread in contiguity with a local soft tissue infection [13, 18].

Regardless of the source, knowing the severity of infection can help to guide management in clinical practice. In 1985, Gachter described an arthroscopic and radiographic classification of knee septic arthritis [15]. This was graded from I to IV with increasing severity ranging from opacity of joint fluid and hyperaemic synovium to infiltration of joint cartilage. Unfortunately of the included studies, only Bohler et al. [6] published data on the severity of their subjects' disease. This limitation is important as some argue that with disease progression and corresponding bone involvement, only an open washout is appropriate [11]. However, it should be noted that Gachter grade is yet to be clinically validated for the management of septic arthritis [39]. Given that the data for this review come from retrospective data bases, the decision‐making process relating to disease severity and subsequent method of washout is uncertain.

Irrespective of grade, effective antimicrobial cover is also of paramount importance. There is both a lack of, and conflicting, evidence surrounding the treatment duration for native joint infection. Regimes can range from 2 to 6 weeks [12], with specific patient circumstances leading to further prolongation in treatment for extended months [30]. As an example of how tenuous the data on treatment duration are, there are some who report no correlation between antibiotic duration and successful infection eradication [27]. It was not possible to comment on the relationship between washout modality and antimicrobial therapy for this review due to its retrospective nature and the heterogenous data available. In light of these factors being inextricably linked, we recommend that future studies make standardisation of the antimicrobial pathway a priority.

### Predictors of successful washout

While there was sufficient evidence to compare arthroscopy to arthrotomy, there is a distinct absence of predictive factors for washout success. The patient, microbiological and surgical factors associated with success are unknown. For example, the optimal type and volume of irrigation fluid required is not clear, neither is the effect of patient selection based on inflammatory markers, pathogen present or co‐morbidities. None of the included studies examined for factors associated with success specifically in arthroscopy or arthrotomy. Instead, predictors for overall successful washout were identified. Dave et al. [10] highlighted the crucial role of time from symptom onset to operation in achieving ultimate source control, irrespective of washout methodology. Other notable risk factors were the presence of methicillin‐resistant staphylococcus aureus [23], higher synovial WBC count [24, 42] and diabetes [45]. These three aforementioned risk factors have previously been identified as predictors for failed surgical management [22].

### All‐cause complication rate and disease severity

Analysis of the four included articles with published data on all‐cause complication rate [7, 14, 25, 45] found a significant risk ratio of 0.75 in favour of arthroscopy. The overall complication rate was 37.3% for all subjects, and a detailed analysis of specific morbidities was reported for all except for Johnson et al. [25]. Using all‐cause complications as a metric has limitations, mainly its inability to differentiate between minor and severe complications. Nonetheless, it does provide context for the inpatient course associated with each washout technique.

Arthroscopy was associated with a significantly lower blood transfusion rate in the three articles reporting on specific complications [7, 14, 45]. Upfill‐Brown et al. [45] noted a significantly improved wound infection rate with arthroscopy; this finding was not borne out in the other aforementioned articles.

Grading the severity of infection is useful when examining complication rates. In 1985, Gachter described an arthroscopic and radiographic classification of knee septic arthritis [15]. Unfortunately of the included studies, only Bohler et al. [6] published data on the severity of their subjects' disease. This limitation is important as some argue that with disease progression and corresponding bone involvement, only an open washout is appropriate [11]. At present, we can only speculate if disease severity impacts the complication rates. However, it would be of interest to know if complication rates in septic arthritis are influenced by washout modality more so than disease severity.

### Length of stay

Eligible articles [7, 25, 45] for length of stay analysis found that there is a significant mean difference of almost 2 days in favour of arthroscopy. From a financial perspective, there is a paucity of evidence to suggest if one treatment modality is more cost‐effective than the other. Kerbel et al. [26] found similar average inpatient costs of approximately $16,000 for arthroscopy and arthrotomy.

Shorter length of stay may also be a surrogate marker of improved function postoperatively, allowing for earlier rehab thereby facilitating discharge. Only two studies [6, 24] reported data on functional ability postindex procedure. This was in the form of knee range of motion with both finding significant results in favour of arthroscopy. This is hypothesised to be due to the minimally invasive nature of arthroscopy which is associated with greater soft tissue integrity. Source control is also an important factor in improving functional status. Persistent infection is associated with inflammation, pannus and subsequent joint contracture, thereby limiting recovery [46]. Regarding these recovery‐based outcomes, the limited evidence to date appears to side with arthroscopy.

### Mortality

The mortality rate associated with septic arthritis is dependent on multiple factors. Increasing age, multiple site septic arthritis, diabetes and intravenous drug usage are all associated with increased mortality [21, 47]. Patients who are admitted to the hospital with an alternative primary diagnosis and develop secondary septic arthritis have an even greater morality rate [1]. Four articles published sufficient data for risk ratio calculation [7, 14, 24, 45]. Overall the mortality rate was 1.6%, and we found no statistical difference in mortality for subjects treated with arthroscopy compared to arthrotomy. The interpretation of these results is limited by the variation in follow‐up time, ranging from 1 to 6 months.

### Future arthroplasty

Another consideration is the long‐term prognosis for patients postnative knee septic arthritis. An associated risk of future arthroplasty has been reported to be up to six times that of the normal population [1]. For cases where patients have been cured of their septic arthritis and present for elective knee arthroplasty at a later date, these candidates are at a greater risk of subsequent prosthetic joint injection compared to subjects with no prior history of native joint infection [5]. This is all the more relevant bearing in mind that prosthetic joint infection remains one of the most common indications for revision knee arthroplasty [35].

### Strengths

The primary strength of this review is the scientific approach taken for data collection and analysis. Both were conducted in line with the PRISMA [40] and Cochrane guidelines [19]. Selected articles were identified using systematic and formulaic algorithms used by the two independent reviewers. Analysis included a risk of bias assessment and consideration for article heterogenicity. Random and fixed effects models were used when appropriate and leave‐one‐out sensitivity analysis identified one study as a potential outlier [45].

Strict inclusion and exclusion criteria were utilised to ensure that included articles were relevant and of suitable scientific standard. We note a potential study for inclusion from Yeo et al. [48] with relevant outcomes, however, given that there is no description for diagnosis of septic arthritis, it was accordingly excluded. Another article by Kerbel et al. [26] reported on our secondary outcomes comparing arthroscopy and arthrotomy and would have been considered for inclusion. However, no comparison of repeat washout rate meant it did not fit our inclusion criteria.

Another strength of this review is the cumulative number of subjects. In total, 17,140 index procedures for septic arthritis were identified. We note previous systematic reviews on this topic [2, 11, 28, 38]; however, ours is the first to include Upfill‐Brown et al.'s [45] 14,365 subjects. In light of limited RCT on this subject, we believe ours to be the most definitive body of evidence to date for comparing arthroscopy to arthrotomy for septic knee arthritis.

### Limitations

The main limitation of this review is the lack of RCTs available for inclusion. During our search, only one RTC by Peres et al. [37] was identified. Subjects were followed for 24 months and a lower reinfection rate was noted with arthroscopy. This article was not included in our review as a result of its different, albeit superior, level of evidence. In keeping with Cochrane guidelines, included articles should be similar enough to be grouped for comparison [19] and the one RTC does not meet this criteria in our review.

The retrospective studies selected had intrinsic flaws in their study design. All articles included in this review were retrospective and overall had a moderate risk of bias. This was primarily due to the subject selection process and selection of the intended treatment modality. Treatment modality was typically decided upon by the operating surgeon, with no elaboration on this decision‐making process. Furthermore, there are clinicians who advocate for planned repeat washouts, especially with a Gachter grade III or IV. According to our definition of successful treatment, planned staged washouts would be deemed as treatment failure. Jaffe et al. [23] were the only study group to explicitly exclude subjects undergoing a planned subsequent washout. The resulting heterogeneity of our results should be acknowledged, and our results need to be interpreted in this context.

The discrepancy in follow‐up time and failure to report on key outcomes should also be noted. The length of clinical follow‐up postindex procedure ranged from 1 [7, 14, 25] to 194 months [10]. One study did not report their follow‐up time period [42]. This disparity makes drawing conclusions on repeat infection rates challenging. Only two studies published data on range of motion postprocedure [6, 24]. With a focus on future prospective trials, we recommend that key secondary outcomes such as postoperative range of motion, length of stay in hospital and complication rates are published to allow for future data extraction and analysis.

## CONCLUSION

In this review, we found there to be no significant difference in repeat washout rate for septic knee arthritis when comparing arthroscopy to arthrotomy. Our review includes 17,140 subjects and contains the largest number of participants in any review of this topic to date. Our finding of equivocal repeat washout rates between arthroscopy and arthrotomy is in keeping with the existing body of evidence.

Regarding our secondary outcomes, we found the all‐cause complication rate to be statistically lower for arthroscopy as was the length of inpatient stay. Mortality rates between the two modalities were comparable. The improved complication rate and decreased length of stay associated with arthroscopy is most likely explainable by the decreased soft tissue injury associated with the minimally invasive technique.

## AUTHOR CONTRIBUTIONS

Study design, data collection and write‐up were performed by Daniel P. McKenna. Peggy Miller contributed with data collection and screening of articles selected for analysis. The concept and topic for this systematic review were devised by Timothy McAleese and May Cleary. Timothy McAleese also performed article screening and review of the draft write‐ups. Timothy McAleese and May Cleary reviewed the draft submission and assisted with the final write‐up.

## CONFLICT OF INTEREST STATEMENT

The authors declare no conflict of interest.

## ETHICS STATEMENT

This systematic review utilised published data from previous studies. In line with local practice, no ethical approval was required for this review. Informed consent was not necessary for this review given that all the data were sourced from previously published literature.

## Data Availability

Data for this review were sourced from the cited articles within the text. Analysis was completed using RevMan 5.6 and source spreadsheets can be made available upon request.
